# Parental Perspectives on Oral Health and Access to Care in Children with Down Syndrome: A Narrative Review

**DOI:** 10.3390/children12050655

**Published:** 2025-05-20

**Authors:** Petra Saitos, Raluca Iurcov, Abel Emanuel Moca, Teofana Bota, Rebeca Daniela Marton, Mihai Juncar

**Affiliations:** 1 Doctoral School of Biomedical Sciences, University of Oradea, 1 Universității Street, 410087 Oradea, Romania; petrasaitos@uoradea.ro (P.S.); teofana.bota@uoradea.ro (T.B.); rebeca.marton@uoradea.ro (R.D.M.); 2 Department of Dentistry, Faculty of Medicine and Pharmacy, University of Oradea, 10 Piața 1 Decembrie Street, 410073 Oradea, Romania; riurcov@uoradea.ro (R.I.); mihaijuncar@uoradea.ro (M.J.)

**Keywords:** Down syndrome, oral health, parents, oral health-related quality of life

## Abstract

**Background/Objectives**: Children with Down syndrome are predisposed to various oro-dental conditions, which can impact their oral-health-related quality of life (OHRQoL) and overall well-being. Given their critical role, parents’ and caregivers’ perceptions are essential for understanding the challenges in oral care access and quality. This narrative review aimed to synthesise the available evidence on parental and caregiver perceptions regarding oral health, OHRQoL, and dental care access for children with Down syndrome. **Methods**: A literature search was conducted in PubMed, Web of Science, and Scopus, covering studies published between January 2015 and January 2025. The search included the keywords “Down syndrome”, “oral health”, “oral health-related quality of life”, “caregivers”, “parents”, “dental care access”, and “special needs children”. Thirteen studies were included after applying eligibility criteria, which selected original research focusing on the target population and themes. **Results**: The findings highlighted that oral health significantly affects the quality of life of children with Down syndrome, influencing their functional, emotional, and social well-being. Caregivers often reported a gap between perceived and clinically observed oral health. Barriers to dental care access included insufficient training of dental professionals, financial constraints, systemic limitations, and perceived biases. Variations across different countries also revealed the influence of cultural and healthcare system factors. **Conclusions**: Oral health plays a critical role in the lives of children with Down syndrome and their families. Strengthening caregiver education, improving communication, validating adapted OHRQoL instruments, and enhancing dental professionals’ training in special needs care are crucial steps to ensure equitable and family-centred oral healthcare.

## 1. Introduction

Children with special health care needs (CSHCNs) represent a distinct population group facing challenges that often go beyond typical pediatric needs due to their diverse physical, developmental, behavioral, and emotional conditions [1]. This category of patients is broadly defined, primarily to encompass the wide range of health-related consequences associated with their conditions [2]. The prevalence of children with special health care needs varies across populations, with some studies reporting rates as high as 25.3% [3], and an overall increasing trend has been noted in recent years [4]. From a pathophysiological perspective, CSHCN are affected by various systemic conditions that disrupt normal physiological processes and impact multiple organs [2]. Oral health is no exception, as children with disabilities are at higher risk of developing oral diseases throughout their lives, negatively affecting their overall quality of life [5]. Poor oral hygiene, periodontal problems, and a higher prevalence of dental caries are among the most commonly reported oral health issues in this population [6]. This is particularly concerning given the increased barriers these children face in accessing dental care. Factors contributing to these barriers include dentists’ reluctance to treat patients with disabilities, dental anxiety, the financial burden of care, and the limited availability of appropriate treatment options [7].

Down syndrome (DS), or trisomy 21, is the most common genomic disorder associated with intellectual disability, caused by the presence of an extra chromosome 21 [8]. The global age-standardized incidence is estimated at 1.21 cases per 100,000 population, with higher rates in Western Europe (2.1 per 100,000) and 1.43 per 100,000 in Eastern Europe, exceeding the global average [9]. Most individuals with DS (95%) have the classic form, while 3% present with translocation DS and 2% with mosaic DS, characterized by a mixture of normal and trisomic cells [10].

Since its first description in 1959 [11], DS has been associated with a wide spectrum of clinical manifestations affecting multiple systems [8]. Neurological features include intellectual disability, sleep apnea, seizures, behavioral disorders, and a higher risk of Alzheimer’s disease [12]. Musculoskeletal issues, such as joint laxity, pes planus, arthritis, and hip instability, are frequently reported [13]. Cardiovascular complications often include congenital heart defects and pulmonary hypertension [14]. Other typical characteristics include muscular hypotonia, short neck with excess nuchal skin, flat facial profile, microcephaly, upward-slanting palpebral fissures, and short digits [15].

Individuals with DS also exhibit several specific oral manifestations. Common mucosal findings include macroglossia, geographic tongue, cheilitis, and recurrent candidiasis [16]. Dental anomalies affecting number, shape, size, and structure are reported up to five times more frequently than in the general population, often accompanied by delayed or atypical eruption patterns [17]. Although individuals with intellectual disabilities generally present with poorer oral hygiene, children with DS tend to have a lower caries experience compared to their peers [18]. This may be related to factors such as microdontia, which creates interdental spacing, bruxism, and higher salivary IgA levels that inhibit bacterial adhesion and neutralize cariogenic factors [19]. In contrast, periodontal disease is highly prevalent in this population, affecting up to 90% of individuals with DS under the age of 30. This increased susceptibility is driven by genetic factors, immune deficiencies, early colonization by periodontal pathogens, and poor oral hygiene practices [20,21,22].

Globally, DS represents a significant public health concern due to the substantial burden it places on families and society. Families of individuals with DS face both economic challenges related to healthcare and emotional burdens, which may negatively impact their quality of life [23]. Their perspective is essential, as they serve as the primary caregivers and advocates for their children [24]. Oral health plays a critical role in the overall well-being of children with DS, who are predisposed to multiple oro-dental conditions [17]. If left unaddressed, these issues can impact nutrition, communication, self-esteem, and, ultimately, the quality of life of both the child and their family [25]. Therefore, it is essential to consider not only the clinical aspects of oral health but also the lived experiences, perspectives, and challenges faced by parents and caregivers in accessing dental care for children with DS.

The aim of this narrative review is to explore and synthesize the current literature regarding the perceptions of parents and caregivers on the oral health of children (0–18 years) with DS. Specifically, this review focuses on how caregivers’ perceptions influence oral-health-related quality of life (OHRQoL) in this population and highlights the barriers and challenges they face in accessing adequate dental care services. By analyzing studies conducted in different cultural and healthcare contexts, this review seeks to identify common patterns, gaps in knowledge, and areas for improvement.

## 2. Materials and Methods

### 2.1. Search Strategy

A narrative review was conducted to explore the perceptions of parents and caregivers regarding the oral health of children with DS, with particular attention to oral health-related quality of life (OHRQoL) and access to dental care. The review process was conducted in accordance with the Scale for the Assessment of Narrative Review Articles (SANRA) guidelines, which provide a framework for ensuring methodological transparency, clarity, and rigor in narrative literature reviews.

The search was carried out in the electronic databases PubMed, Web of Science, and Scopus. A structured search strategy using Boolean operators (AND, OR) was employed to combine relevant keywords, including: “Down syndrome”, “oral health”, “oral health-related quality of life”, “caregivers”, “parents”, “dental care access”, and “special needs children”. The following search string was used in all databases: (“Down syndrome” AND “oral health” AND (“oral health-related quality of life” OR “OHRQoL”) AND (caregivers OR parents) AND (“dental care access” OR “special needs children”)).

Articles published between January 2015 and January 2025 were considered. The time frame of January 2015 to January 2025 was selected to identify the most recent and relevant studies on the topic, particularly given the growing attention over the past decade to the impact of oral health on quality of life, both in the general population and among individuals with disabilities. While foundational studies published prior to this period are acknowledged, this narrative review aimed to prioritise studies that reflect current clinical practices, contemporary parental perceptions, and the challenges currently faced by families within today’s healthcare systems. This approach was intended to enhance the practical relevance and applicability of the findings to current healthcare contexts. The search was conducted between 1 April 2025 and 10 April 2025.

### 2.2. Study Selection and Eligibility Criteria

Two authors independently performed the literature search and screened all articles by title and abstract, selecting those most relevant to the objectives of this narrative review.

The inclusion and exclusion criteria were established to ensure the review focused on original research providing empirical evidence on caregiver perspectives, OHRQoL, and dental care access in children with DS, published within the selected time frame and accessible in English.

Studies were included if they met the following inclusion criteria: (1) original research articles; (2) studies focusing on children with ages up to 18 years diagnosed with DS; (3) studies investigating parental or caregiver perceptions regarding oral health, oral health-related quality of life (OHRQoL), or access to dental care; (4) articles published between January 2015 and January 2025; and (5) articles written in English.

Studies were excluded if they were systematic reviews, meta-analyses, case reports, studies focusing exclusively on adults, or articles not available in full text. Additionally, grey literature, including theses, conference proceedings, and non-peer-reviewed sources, was not included in this review.

**Figure 1:**
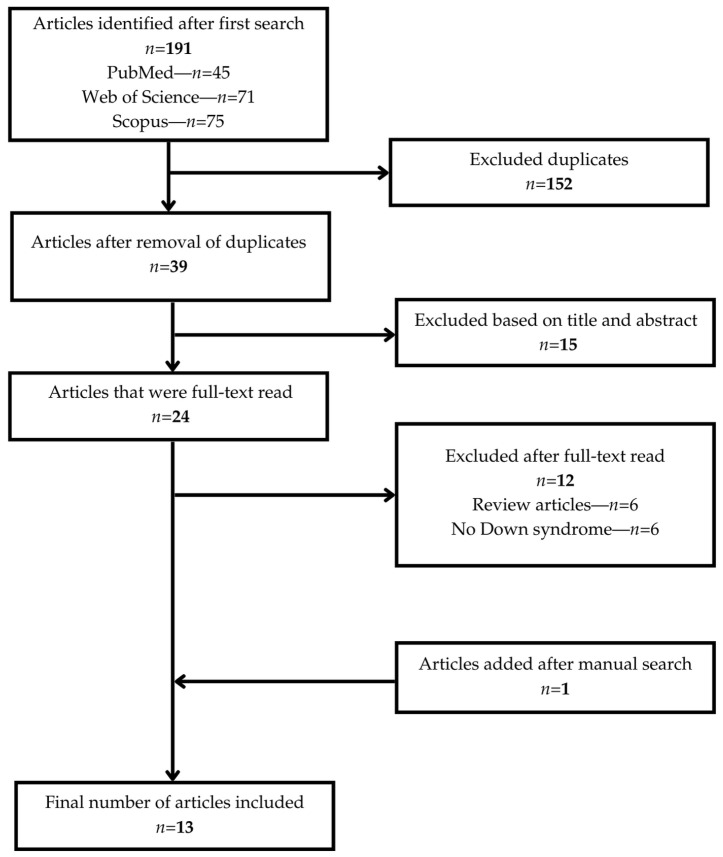
Study selection process.

The initial search retrieved 191 articles from PubMed (*n* = 45), Web of Science (*n* = 71), and Scopus (*n* = 75). After removing 152 duplicate records, 39 articles remained. Following the screening of titles and abstracts, 15 articles were excluded. A total of 24 articles were assessed for full-text eligibility. Of these, 12 articles were excluded: 6 were review articles and 6 did not address DS. One additional article was identified through a manual search. Ultimately, 13 articles were included in this narrative review. The study selection process is illustrated in Figure 1, presented in a PRISMA-style flowchart. The 13 studies included in this narrative review are as follows:

- Faria Carrada et al. (2020)—Brazil [26]
- Scalioni et al. (2018)—Brazil [27]
- Essam et al. (2025)—Egypt [28]
- Nqcobo et al. (2019)—South Africa [29]
- AlJameel et al. (2020)—Saudi Arabia [30]
- AlJameel et al. (2021)—Saudi Arabia [31]
- Onishi et al. (2025)—Japan [32]
- Stensson et al. (2021)—Sweden [33]
- Stensson et al. (2022)—Sweden [34]
- Kalyoncu et al. (2018)—Turkey [35]
- Wan Roselan et al. (2023)—Malaysia [36]
- Stein Duker et al. (2020)—United States [37]
- Descamps and Marks (2015)—Belgium [38].

## 3. Narrative Synthesis

### 3.1. Oral Health-Related Quality of Life (OHRQoL) and Down Syndrome

Quality of life (QoL) is a multidimensional concept describing an individual’s general state of well-being in relation to their values, environment, and the cultural and social context in which they live [39]. The World Health Organization defines QoL as “a person’s perception of their position in life in the context of the culture and value systems in which they live and in relation to their goals, expectations, standards and concerns” [40]. In order to assess quality of life, various instruments have been developed, either as generic tools for the general population or as disease-specific instruments for targeted groups [41].

Recognizing that oral health may significantly affect individuals’ well-being, the concept of oral health-related quality of life (OHRQoL) has gained increased attention in recent decades. OHRQoL has important implications not only in clinical dental practice, but also in oral health research [42]. By using validated tools, researchers can investigate how oral health influences the biological, psychological, social, and cultural aspects of patients’ lives [42]. The most frequently used tools for adults include the Geriatric Oral Health Assessment Index (GOHAI), the Dental Impact Profile (DIP), and the Oral Health Impact Profile (OHIP) [43]. In children, the most commonly applied instruments are the Early Childhood Oral Health Impact Scale (ECOHIS), the Scale of Oral Health Outcomes for 5-Year-Old Children (SOHO-5), and the Child Oral Health Impact Profile (COHIP) [44]. Although these instruments are useful for capturing a patient’s subjective experience, direct self-reporting may be limited or unreliable in children with DS due to intellectual disabilities. Therefore, parental or caregiver proxy reporting becomes essential for assessing OHRQoL in this population [45]. Parents of children with DS often serve as the primary interpreters of their children’s needs and daily experiences. Their perceptions provide critical insights into how oral health conditions affect quality of life, including aspects such as pain, functional limitations, emotional distress, social interaction, and access to care. A growing body of research has focused on parent-reported OHRQoL to better understand these specific challenges and to inform clinical practice and public health policies.

A key study conducted by Faria Carrada et al. (2020) aimed to evaluate caregivers’ perceptions of the impact of various oral conditions on the OHRQoL of children with DS, compared to children from the general population, in a sample from Juiz de Fora, Brazil. The study employed the Brazilian short version of the Parental–Caregiver Perceptions Questionnaire (P-CPQ), which includes 13 questions and was originally validated for caregivers of children aged 11–14 years. However, it has also been applied in research involving broader age ranges, including children older than 14 years. The final sample consisted of 288 caregivers, 144 for children with DS and 144 for neurotypical children aged 4 to 18 years. In addition to completing the questionnaire, the children underwent clinical evaluations, including caries experience (DMFT and PUFA indices), malocclusion (Dental Aesthetic Index), and oral hygiene (Visible Plaque Index and Gingival Bleeding Index). However, the published results primarily reported findings based on the PUFA index, with no detailed DMFT data provided. The results showed that caregivers of children with DS reported a more negative perception of the impact of oral health on their child’s OHRQoL, particularly in the domain of functional limitations. However, for oral symptoms, caregivers of children from the general population reported more negatively—likely due to the children with DS being less able to express discomfort. The study reported that children with DS who presented with visible plaque were 1.48 times more likely to be perceived by caregivers as having reduced OHRQoL, and those with untreated dental caries (as measured by PUFA) had a 1.72 times higher prevalence of negative perceptions. These findings underscore the importance of parental awareness of functional impacts while acknowledging that symptom perception may vary due to cognitive and communicative factors [26]. The study also reinforced the relevance of the P-CPQ instrument, initially proposed by Jokovic et al. in 2003 [46], with 31 items covering four domains: oral symptoms, functional limitations, emotional well-being, and social well-being. The short Brazilian version with 13 items, used in this study, was proposed in 2009 [47].

A recent study by Essam et al. (2025) examined the OHRQoL of children with DS aged 4–14 in Cairo, Egypt, from the perspective of their parents [28]. In addition, the study evaluated home oral hygiene practices. Unlike the Brazilian study, the authors used an Arabic translation of the Allison and Lawrence (2005) questionnaire [48], and to evaluate hygiene routines, parents completed a second questionnaire developed by Al-Hussyeen and Al-Sadhan (2006), also in Arabic [49]. Most parents (80.9%) reported a positive perception of their child’s OHRQoL, while only 19.1% had a negative view. However, important hygiene gaps were identified—for example, 61.35% of respondents brushed their children’s teeth with only water (without toothpaste), and only 38.14% used a toothbrush for oral hygiene. When assessing their children’s oral health status, 53.6% selected “good”, while 13.4% reported “poor” oral health [28]. This mismatch between hygiene practices and perceived oral health may reflect a lack of awareness of the implications of oral conditions, particularly given that children with DS may exhibit reduced or delayed pain responses [50]. Despite this, oral diseases can progress rapidly, making parental education critical to prevention [51].

Although caries management has improved significantly in recent years, dental caries remains a major global public health issue [52]. Untreated caries can lead to infectious complications [53], which often require complex interventions under general anesthesia—particularly in children with intellectual disabilities [54]. To avoid such outcomes, early parental education and oral health promotion are essential [55]. In a 2019 study conducted in Johannesburg, South Africa, researchers evaluated the impact of dental caries on OHRQoL in children with special needs, the majority of whom had DS. A short version of the Parent–Caregiver Perception Questionnaire (P-CPQ) was used to assess OHRQoL, covering four domains: oral symptoms, functional limitations, emotional well-being, and social well-being. Clinical assessments of the children were also conducted. The sample included 150 caregivers, of whom 41% were caring for children with DS. Most respondents were women (94.7%), predominantly mothers (87%), highlighting the central role of mothers in caregiving. However, the study did not specify whether all caregivers lived with the child on a daily basis, which may influence the accuracy of their perceptions. Among these caregivers, 91% reported that oral conditions negatively impacted their child’s quality of life. Children with caries in the primary dentition had significantly higher scores in oral symptoms, functional limitations, and overall OHRQoL. Interestingly, when asked about the effect of oral conditions on overall well-being, 60.7% of caregivers stated that their child’s general health was not affected. Most caregivers (56.7%) rated their child’s oral health as average, and only 12% considered it poor. Nevertheless, 30.6% of children with DS had caries in their primary dentition, and 92.8% of those lesions were untreated. These findings suggest that caregivers may underestimate the influence of oral health on general well-being, potentially due to a lack of awareness. As a result, the authors recommend expanding oral health education not only for caregivers but also for educators, particularly in schools for children with special needs and in maternal and child health centers [29].

Despite the high burden of untreated dental caries and the apparent gaps in awareness among caregivers, most existing studies address perceptions of both male and female caregivers. However, on a global scale, women remain the primary caregivers and are most often responsible for the informal care of family members [56]. While motherhood can be deeply rewarding, it is also associated with increased mental and physical strain, a greater caregiver burden, and higher levels of psychological distress [57,58]. These factors make the maternal perspective particularly valuable when assessing perceptions of oral health. Among the reviewed studies, only one focused exclusively on mothers of children with DS, exploring both their perceptions of oral health and the broader impact on their children’s well-being. Conducted by AlJameel et al. (2020) in Riyadh, Saudi Arabia, the study involved in-depth interviews with 20 mothers of children aged 12 to 18 years. Unlike the other studies, this qualitative research formed the basis for the development of a new questionnaire aimed at capturing the influence of oral health on quality of life in children with DS. Although the majority of mothers initially rated their children’s oral health as good, they also frequently reported issues such as tooth decay, dental pain, and functional limitations, especially related to chewing and speech. Interestingly, many mothers were initially reluctant to attribute changes in their child’s mood or behavior to oral discomfort. However, with further reflection, they acknowledged that during episodes of dental pain, their children exhibited increased irritability, reduced laughter, crying, or withdrawal, signs that oral health was indeed influencing quality of life in subtle but meaningful ways [30].

The importance of validating new instruments tailored to assess OHRQoL in children with DS was emphasized by AlJameel and AlKawari (2021), who applied the OH-QOLADS (Oral Health-Related Quality of Life for Children with Down Syndrome) questionnaire in a sample from Riyadh, Saudi Arabia. The study included 63 parents of children aged 10–14 years with DS. Regarding the impact of oral health on the child’s quality of life, 65.1% of respondents stated that oral health had no impact, while 34.9% reported some level of influence. Notably, 54% of parents said their children had experienced dental pain (with 22.2% reporting severe pain), and 41% reported that eating habits were affected by oral health issues. Interestingly, the study also evaluated the impact of the child’s oral health on the family’s quality of life, using the same questionnaire. A total of 46% of respondents believed that their child’s oral health affected the family’s emotional well-being, with common issues cited including frustration, worry, and self-blame [31].

The findings from these four studies clearly demonstrate that oral health significantly affects the quality of life of children with DS, as well as the emotional and social well-being of their families. A potential underestimation of oral symptoms, due to delayed pain response and communication difficulties, further underscores the essential role of caregivers in identifying early signs of oral disease and maintaining adequate hygiene. In this context, the validation and application of adapted OHRQoL assessment tools are vital for a more accurate understanding of this population’s needs and for the development of personalized, family-centered clinical and educational interventions.

### 3.2. Parental and Caregiver Perceptions of Oral Health in Children with Down Syndrome

Globally, over one billion people live with some form of disability, accounting for approximately 15% of the world’s population. Disability often imposes a significant burden, both on the affected individual and on the family members who provide daily care [59]. People with disabilities frequently experience health inequities, often rooted in systemic discrimination and social exclusion, which expose them to a higher risk of medical conditions than the general population [60]. For individuals with DS, health risks are compounded by common comorbidities such as hearing and vision impairments, congenital heart defects, thyroid disorders, as well as numerous oral health problems, including dental anomalies, malocclusion, periodontal disease, and untreated caries [61]. In this context, caring for a child or adult with DS can become especially challenging for families, particularly when financial, logistical, or emotional resources are limited and access to healthcare services is restricted [59].

Given the central role that parents and caregivers play in the daily care, support, and education of individuals with DS, understanding their perception of oral health is essential in order to tailor dental services to meet the actual needs of this population [62]. Parental perceptions influence not only daily oral hygiene behaviors, decisions to seek dental care, and adherence to treatment, but also the recognition and interpretation of oral symptoms, which, given the limited communication ability of many individuals with DS, depend almost entirely on the caregiver [62].

To better understand how parents and caregivers perceive the oral health of children with DS, several recent studies conducted across diverse cultural and geographic contexts have been analyzed. These studies offer complementary perspectives on parental knowledge, attitudes, and behaviors, while also highlighting the clinical, socio-demographic, and emotional factors shaping these perceptions.

A study conducted in Japan and published in 2024 examined parents’ perceptions of general health, oral health, and dental care for children with DS. Based on an online questionnaire, the study explored aspects such as dental visits, oral hygiene habits, and the presence of oral pathologies. The questionnaire used in the Japanese study was based on previously published, internationally recognized instruments, including the validated Oral Assessment in Down Syndrome (OADS) questionnaire. It was culturally adapted to Japanese and pilot-tested before data collection, enhancing its content validity. While 43% of parents rated their child’s oral health as good or very good, 57% described it as moderate or poor. Among children under the age of four, 19% had never visited a dentist, mainly due to the belief that they were too young, lack of knowledge about when the first visit should occur, or difficulty finding a dentist willing to treat children with disabilities. Most parents reported being satisfied with their child’s dentist, valuing qualities such as reassurance, specialization in disability care, empathy, and efforts to engage the child. In contrast, dissatisfaction stemmed from a perceived lack of enthusiasm, inadequate communication with parents, failure to reassure the child, and long waiting times for appointments [32]. These findings underscore the importance of the parent–dentist relationship, especially given that dentists themselves often report a lack of training in disability care as a barrier to treating children with special needs [63]. Moreover, few dentists have ever treated a child with DS, and dental anxiety in children was frequently cited as a factor that discouraged families from seeking routine care [64].

Anxiety and lack of cooperation often increase the likelihood of treatment under general anesthesia [65]. In a study by Stensson et al. (2021), which used a questionnaire that had been previously developed and validated in Belgium, based on internationally recognized instruments, it was noticed that from a sample of 101 parents, 24 children had received dental treatment under general anesthesia, 12 had been sedated with N_2_O, and 19 with rectally administered benzodiazepines. Surprisingly, the most common procedures performed under general anesthesia were basic clinical examinations and dental cleanings, procedures ideally conducted without sedation. Nonetheless, most parents assisted their children during brushing, had taken them to the dentist within the past six months, and reported being satisfied with the dentist, valuing traits such as patience, empathy, involving the child, and allowing extra time [33].

In Brazil, a study involving a 20-item questionnaire combined parental perceptions with clinical evaluations. While positive perceptions were predominant in terms of eating, communication, oral habits, and oral symptoms, nearly 50% of responses reflected negative views, suggesting a close divide [27]. This discrepancy may be due to limited parental knowledge of oral health [55]. To address this, Kalyoncu et al. (2018) conducted a study in Türkiye to assess the knowledge and attitudes of families with children who have DS. Among the 103 respondents, 24.3% had never taken their child to a dentist, and 11% had never received any formal information regarding oral hygiene. Preventive dental care was rare, and most treatments provided were reactive (e.g., extractions) rather than preventive [35].

In this context, oral health education and promotion are essential for reducing the incidence of dental caries and periodontal disease, and for improving the quality of life for individuals with DS [66]. This effort must include the education and training of disability support staff, caregivers, and the patients themselves [67,68]. Furthermore, dental professionals need to be equipped with specific skills and confidence to effectively manage patients with special needs [69]. Based on these identified needs, the study by Wan Roselan et al. (2023) aimed to evaluate the oral health care experiences and practices of parents of children with DS, with the ultimate goal of developing a comprehensive oral health promotion programme. Conducted in Malaysia, the study surveyed 75 parents, 88% of whom were mothers, using a validated bilingual questionnaire assessing three domains: oral health experiences, oral hygiene practices, and dietary habits. Although most parents (79.7%) reported that their child’s oral and gingival health was good, the study found no significant correlation between experience scores, practice scores, and perceived oral health. Furthermore, parental education level did not significantly influence oral hygiene practices, suggesting that factors such as social support or access to information may play a more important role. Most parents (77%) reported brushing their child’s teeth twice a day, and 88% stated that they used fluoride toothpaste, indicating a generally positive attitude towards daily oral care. Additionally, 50% of parents reported limiting their child’s consumption of sweets, and 78.4% had taken their child to the dentist within the past year, reflecting encouraging preventive care practices. The findings reinforce the need for tailored educational programmes that both reinforce existing behaviors and address persistent barriers to accessing adequate dental care for children with DS [36].

The reviewed studies highlight that parental and caregiver perceptions play a critical role in shaping the oral health outcomes of children with DS. While many caregivers report positive attitudes and practices, discrepancies between perceived and clinically observed oral health underscore the need for increased awareness and education. Strengthening caregiver education and equipping dental teams with the necessary skills to manage patients with disabilities are essential steps towards improving both oral health and quality of life for children with DS and their families.

### 3.3. Dental Care Experiences and Access to Services for Children with Down Syndrome

Oral care is narrowly defined as the cleaning of teeth, the mouth, and dentures, and more broadly as the maintenance of oral functions, including chewing and swallowing training, as well as temporomandibular joint exercises, and is a concept that has gradually expanded over time [70]. Despite the significant impact of oral health on general health and overall well-being, dental treatments covered by national health systems are often restricted to certain procedures and specific age groups [71]. Moreover, for individuals with disabilities, access to dental care is even more limited, with barriers including financial constraints, dentists reluctance to treat this patient group, systemic challenges, and patient-related factors [72].

To explore how parents of children with DS perceive their children’s needs in dental healthcare settings, Stensson et al. (2022) investigated five categories of needs identified by Swedish parents. These included the lack of continuity in dental care, insufficient knowledge and experience among dental professionals in treating children with DS, and inadequate communication with these patients. Parents highlighted the need for acclimatization visits to familiarize children with the dental environment and advocated for their children to be treated similarly to typically developing children. Many Swedish parents perceived a bias in the dental treatment provided to children with DS. This included assumptions that their child would be unable to cooperate, leading to incomplete or avoided treatments. Some parents also expressed concerns that dental professionals considered aesthetics and comprehensive dental care less important for children with DS compared to typically developing children, reinforcing feelings of inequality and exclusion [34].

Similar difficulties were reported by Stein Duker et al. (2020) in a U.S.-based study using the Dental Care in Children Survey. Although 60% of respondents stated that their children regularly attended a dental clinic, and 35% had a dentist specializing in the treatment of children with DS, nearly 80% found it moderately to extremely difficult to locate a dentist willing to provide care for their child. Furthermore, 61% of parents reported that their child had been refused care by a dental provider. Reasons for refusal included a lack of training, the extended time required for procedures, and behavioral challenges posed by the children [37].

Regarding access to dental care, Descamps and Marks (2015) conducted a study on a population in Belgium [38]. According to World Health Organization data, the Belgian national healthcare system covers the costs of routine and preventive dental care, essential curative oral health care, and advanced curative treatments [73]. This coverage may explain why 89% of children with DS in the cited study had visited a dentist at least once in the past year. However, 68% of parents preferred taking their children to a private dental clinic, while only 7% chose a dentist specialized in treating children with special needs [38].

The studies reviewed highlight the major barriers faced by children with DS in accessing adequate dental care. Lack of continuity of care, limited experience among dental professionals, and financial or systemic obstacles significantly impact service accessibility. Even in countries where preventive dental care is publicly funded, parents often prefer private practices, signaling a pressing need for more personalized approaches, effective communication, and specialized training for dental professionals.

## 4. Discussion

Considering the increasing number of CSHCN reported in recent years [4], as well as the rising trend observed in Europe for children with DS, as highlighted in the EUROCAT surveillance report [74], and the higher prevalence of dental anomalies and oral pathologies in this population [16,17,18,19,20,21,22], investigating parental and caregiver perceptions of the oral health of children with DS is essential. This narrative review specifically aimed to explore how parents and caregivers perceive the impact of oral health on the quality of life of children with DS, as well as the challenges related to accessing appropriate dental care.

The findings of this analysis highlight the significant role that oral health plays in the daily lives of children with DS and their families [26,28,29,30,31]. Although existing literature suggests a lower prevalence of dental caries among individuals with DS [18], periodontal disease, dental anomalies, and functional difficulties such as chewing and speech are common [16,17,20]. These issues affect not only physical health but also eating, communication, social interaction, and the development of self-esteem [25]. Unfortunately, studies show that parents, despite being involved and attentive, may not always recognize the signs of these problems. Communication challenges or the subtle expression of pain in children with DS can lead parents to believe that everything is fine when it is not [26,28,29,30,31]. This underlines the importance of providing parents and caregivers with the support they need to identify oral health problems early and to learn how to prevent or manage them effectively, with the help of dental professionals [75].

Although healthcare services are theoretically meant to be accessible and inclusive for all, the reality suggests otherwise. Children with DS and their families often face additional barriers that place them at a disadvantage compared to other children. A consistent theme across the reviewed studies is the difficulty in accessing dental care tailored to the needs of children with DS. Whether in countries with well-developed public healthcare systems or in resource-limited settings, parents report similar challenges: long waiting times, high costs, and a shortage of dental professionals trained to work with this population [34,37,38]. In some cases, parents have even reported being denied treatment, further deepening feelings of exclusion and discrimination [37]. These realities raise serious concerns about whether health equity is truly being achieved, especially regarding access to quality dental care for children with disabilities.

An important observation from the reviewed literature is how different healthcare systems approach the care of children with DS. While parents in countries such as Sweden and Belgium report that basic care is covered by public health services, they still encounter difficulties in finding specialists trained to treat their children [34,38]. In other regions, such as parts of Asia, Africa, or Eastern Europe, the lack of specialized public services forces families to rely almost entirely on private care, significantly limiting access for those with fewer financial resources [35,36,71]. The recurring reports of service refusals, high costs, and lack of trained professionals suggest that health equity remains more of an ideal than a lived reality for many families. This is further supported by the 2023 WHO and UNICEF Global Report, which highlights that millions of children with disabilities continue to be excluded or overlooked in health and education policies [76,77].

In Romania, dental services for children with disabilities are currently limited, being almost exclusively available in private practices, which significantly reduces access for families with limited financial resources. Although there are dedicated practitioners and local initiatives, these efforts are not part of a coherent national strategy and lack support through clear public policies or national training programs [78]. At the European level, significant disparities are evident between member states, with some countries implementing prevention programs and policies dedicated to individuals with special needs, while others, including Romania, continue to lag behind [71]. This lack of uniformity in policies and access to specialized services underscores the urgent need for coordinated action at both national and European levels to ensure real equity for all children, regardless of their specific needs.

This narrative review has several limitations that should be acknowledged. First, the selection of studies was limited to articles published between 2015 and 2025, which, although allowing the focus on recent and relevant data, excluded older studies that might have provided valuable historical or longitudinal perspectives. Additionally, only articles written in English were included, which may limit the generalizability of the findings to other linguistic or cultural contexts. Furthermore, the review predominantly reflects the perceptions of mothers. The experiences of fathers, grandparents, siblings, or professional caregivers are much less documented, despite their important roles in supporting the oral health of children with DS. Future studies should aim to explore these diverse perspectives to provide a more comprehensive understanding of the real challenges and needs of families. Finally, as a narrative review rather than a systematic or quantitative analysis, this synthesis does not provide statistical measurements of the reported effects but offers instead a qualitative interpretation of the themes identified in the literature.

Despite these limitations, this analysis provides a valuable overview of parents’ and caregivers’ perceptions regarding the oral health of children with DS. Future research should include studies that explore in greater depth the experiences of fathers and other caregivers, evaluate the impact of educational interventions on families, and assess the effectiveness of public health policies dedicated to individuals with special needs. Additionally, multicenter studies conducted in diverse cultural and geographical contexts are needed to ensure broader and more internationally applicable insights. Such efforts could contribute to the development of more equitable and better-adapted interventions and policies that meet the real needs of children with disabilities and their families.

## 5. Conclusions

In conclusion, this narrative review highlights the significant impact of oral health on the quality of life of children with DS and the multiple barriers that families face in accessing appropriate care. Addressing these challenges requires coordinated efforts to strengthen caregiver education, improve dental professionals’ training, and promote inclusive, family-centred oral health strategies that ensure equitable access for all children.

## Data Availability

Not applicable.
